# Preterm neonatal mortality in China during 2009–2018: A retrospective study

**DOI:** 10.1371/journal.pone.0260611

**Published:** 2021-12-08

**Authors:** Xue Yu, Chunhua He, Yanping Wang, Leni Kang, Lei Miao, Jian Chen, Qihui Zhao, Xiaona Huang, Jun Zhu, Juan Liang, Qi Li, Meixian Wang, Hanmin Liu

**Affiliations:** 1 National Office for Maternal and Child Health Surveillance of China, West China Second University Hospital, Sichuan University, Chengdu, Sichuan, China; 2 Key Laboratory of Birth Defects and Related Diseases of Women and Children of the Ministry of Education, West China Second University Hospital, Sichuan University, Chengdu, China; 3 Department of Pediatrics, West China Second University Hospital, Sichuan University, Chengdu, China; 4 Department of Pediatrics, Meishan Maternal and Child Care Hospital, Chengdu, China; 5 Department of Pediatrics, Pidu Maternal and Child Care Hospital, Chengdu, China; 6 Health, Nutrition and Water, Sanitation & Hygiene, UNICEF China, Beijing, China; Center of Pediatrics, GERMANY

## Abstract

In this retrospective analysis, we aimed to analyze the epidemic characteristics of neonatal mortality due to preterm birth at 28–36 weeks gestation in different regions from 2009 to 2018. Data were obtained from China’s Under-5 Child Mortality Surveillance System (U5CMSS). The χ^2^ trend test, Poisson regression and the Cochran-Mantel-Haenszel method were used in this study. We found that 51.3%, 42.0% and 44.5% of neonate deaths were preterm infants, and immaturity was mainly attributed to 60.1%, 64.1% and 69.5% of these deaths, in the eastern, central and western regions, respectively. The preterm neonatal mortality rate due to immaturity dropped from 149.2, 216.5 and 339.5 in 2009 to 47.4, 83.8 and 170.1 per 100 000 live births in 2018, giving an average annual decline rate of 12.1%, 11.6% and 6.3% in the eastern, central and western regions, respectively, during the studying period. The relative risk of preterm neonatal mortality due to immaturity were 1.3 and 2.3 for the central regions and western regions in 2009–2010, ascending to 2.2 and 3.9 in 2017–2018. The proportion of preterm neonatal deaths with a gestational age <32 weeks was highest among the eastern region. There were significantly more preterm neonatal infants who were not delivered at medical institutions in the western region than in the eastern and central regions. The preterm infant, especially with gestational age <32 weeks, should receive the most attention through enhanced policies and programs to improve child survival. Priority interventions should be region-specific, depending on the availability of economic and healthcare resources.

## Introduction

Globally, an estimated 2.5 million newborns died in the first month of life—approximately 7 000 every day, accounting for 47% of all under-five deaths in 2018. Between 1990 and 2018, the global annual neonatal mortality rate decreased by 51%, from 37 deaths per 1000 live births to 18 deaths per 1 000 live births. However, during this period, the global annual mortality rate among children under 5 years of age decreased by 58%, from 93 deaths per 1 000 live births to 39 deaths per 1 000 live births [1]. Newborn deaths are decreasing at a slower rate than are under-5 deaths and maternal deaths [2]. Neonates predominantly die because of preterm birth, intrapartum-related complications and infections, which differ from the causes of death of older children [3].

Preterm birth (before 37 completed weeks of gestation) (PTB) continues to be one of the leading causes of neonatal morbidity and mortality worldwide. Worldwide, approximately 3.1 million babies per year dying as a direct result of preterm birth [4]. In addition to its significant contribution to mortality, the effect of preterm birth among some survivors may continue throughout life, impairing neurodevelopmental functioning by increasing the risk of cerebral palsy, learning impairment and visual disorders and affecting long-term physical health with a higher risk of non-communicable disease [5]. Preterm birth is associated with significant costs to health systems, and families of preterm newborns often experience considerable psychological and financial hardship [6–9]. Therefore, preterm birth is one the largest single conditions in the Global Burden of Disease analysis given the high mortality and the considerable risk of life long impairment [10].

Worldwide, an estimated 10.6% of all births are preterm, and regional preterm birth rates range from 13.4% in North Africa to 8.7% in Europe [11]. Over the last 20 years, most countries have shown an increase in the preterm birth rate. In industrialized countries, the rate has been increasing by 19.4% since the early 1980s [12]. In Latin America, the preterm birth rate increased from 7.7% in 1990 to 8.4% in 2010 [13]. The proportion of neonatal deaths in preterm infants shows a corresponding increase. In 2018, complications of preterm birth accounting for approximately 16% of child deaths in children younger than 5 years, and 35% of deaths among newborn babies [1]. The United Nations (UN) summit on sustainable development formally adopted the 2030 agenda for sustainable development in 2015, the SDG3’ goal is to end preventable neonatal (complications due to prematurity, intrapartum-related deaths including birth asphyxia and neonatal infections) by 2030, with all countries aiming to reduce neonatal mortality to at least as low as 12 deaths per 1,000 live births [14]. Hence, addressing the global burden of preterm birth is crucial to achieving SDGs 3.2 and for reducing preterm-related neonatal and child mortality [11, 15]

China represents a unique example of success in achieving the MDG 4 target of reducing the under-5 mortality rate by two-thirds between 1990 and 2015 and is also one of approximately only a dozen countries that achieved a faster decline in mortality in neonates than in children 1–59 months since 1990 [16, 17]. However, approximately 73,000 neonates died in China in 2018, accounting for about 3.0% of the total newborn deaths worldwide, ranking sixth in the world [1]. And according to the NMR data from World Bank, the NMR of China still lags behind that in developed countries (such as the NMR in Australia was 2.3 deaths per 1000 live births, and Germany was 2.2 deaths per 1000 live births) [18]. Moreover, the number of preterm births in China ranks second in the word, accounting for 7.8% of global preterm births, which is just lower than India (23.4%) [11]. In this study, we used data from China’s Under-5 Child Mortality Surveillance System (U5CMSS) to investigate the epidemiology of preterm neonatal mortality during 2009–2018. Our hope is that our study will provide some information for the development of timely and effective preterm infant intervention programs to reduce neonatal mortality.

## Materials and methods

### Data source

**Fig 1:**
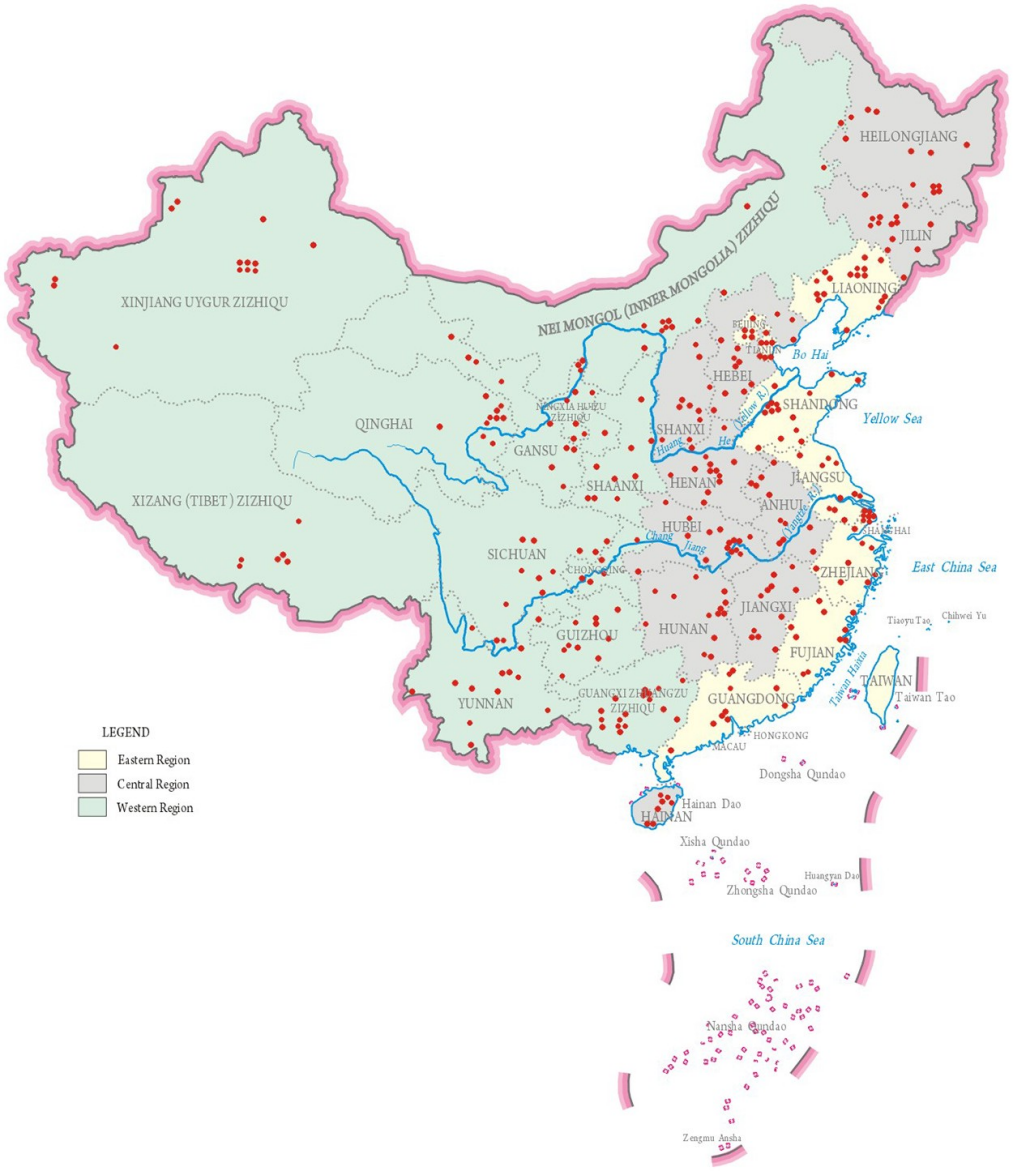
China’s under-5 child mortality surveillance system site map.

This study used data from the U5CMSS, a population-based surveillance system collecting vital statistics on levels and causes of child mortality. Beginning in 2009, the U5CMSS covered a total population of approximately 47.1 million individuals across 334 representative sites, of which 124 are urban districts and 210 are rural counties, in 31 provinces in mainland China. Based on geographical locations and economic conditions, the 31 provinces were divided into eastern, central, and western regions (Fig 1). To reduce the risk of underreporting and to enhance the quality of the data, there are standard procedures in the surveillance network for data collection, reporting, auditing and quality control, which has been described previously [19].

### Statistical analysis

In addition to analyzing the epidemic characteristics of preterm neonatal mortality across the entire study period of 2009–2018, the results were calculated in 2-year intervals to obtain robust estimation. Annual numbers of preterm neonatal deaths and live births were adjusted by a 3-year moving average under-reporting rate based on annual national data quality control results. Comparisons of the proportions of preterm neonatal deaths, the differences in causes of death and preterm neonatal characteristics were measured by the χ^2^ trend test. Trend analysis of preterm neonatal mortality due to immaturity was performed by Poisson regression. The average annual decline rate (AADR) of preterm neonatal mortality due to immaturity and 95% CIs were calculated by Poisson regression. The relative risk (RR) among different regions adjusted by residential areas and the 95% CI were calculated using the Cochran-Mantel-Haenszel method. All statistical analyses in this study were calculated using SASV.9.4 (SAS Institute, Cary, North Carolina, USA). A statistically significant difference was defined as a = 0.05.

### Ethics approval and consent to participate

This study was approved by the Ethics Committee of West China Second University Hospital, Sichuan University, China.

### Patient and public involvement

Patients and members of the public were not involved in the design of this study.

## Results

In 2009–2018, a total of 4 136, 6 744 and 13 835 neonatal deaths were registered in the U5CMSS in the eastern, central and western regions, of which 2123 (51.3%), 2834 (42.0%) and 6151 (44.5%) were preterm infants, respectively. Among the three geographic regions, the percentage of preterm infants among all neonatal deaths was highest along the eastern region between 2009 and 2018. The proportion of preterm infants in neonatal deaths increased from 50.4%, 38.9% and 38.5% in 2009 to 53.1%, 48.4% and 47.9% in 2018, with a significant increase of 5.4%, 8.0% and 24.2% in the eastern, central and western regions, respectively, over the past 12 years (P < 0.05) (Table 1).

**Table 1:** The number of live births, neonatal deaths and preterm infants among neonatal deaths among the three regions in China 2009–2018.

	Eastern region				Central region				Western region			
	Live births	ND	PI	%	Live births	ND	PI	%	Live births	ND	PI	%
2009	106130	467	235	50.3	123119	1010	393	38.9	135205	1622	625	38.5
2010	113587	534	251	47.0	127596	944	365	38.7	147298	1576	655	41.6
2011	117061	457	216	47.3	129412	932	378	40.6	150555	1581	630	39.8
2012	131417	447	228	51.0	136125	858	383	44.6	160357	1539	664	43.1
2013	127757	447	242	54.2	131807	685	285	41.6	163984	1476	659	44.6
2014	135413	379	227	59.9	128825	580	246	42.4	167372	1456	666	45.7
2015	144904	377	203	53.8	120952	472	184	39.0	160407	1315	639	48.6
2016	151508	379	185	48.8	125481	439	195	44.4	166099	1113	574	51.6
2017	167632	369	185	50.1	130409	456	226	49.6	178870	1252	606	48.4
2018	140457	281	149	53.0	111598	368	178	48.4	153177	904	433	47.9
total	1335866	4136	2123	51.3	1265324	6744	2834	42.0	1583324	13835	6151	44.5

ND, neonatal death; PI, preterm infants among the neonatal deaths.

The leading three causes of preterm neonatal deaths among the three geographic regions were immaturity, congenital abnormalities, and birth asphyxia, which accounted for approximately 80% of the total preterm neonatal deaths. Immaturity, the leading cause of preterm neonatal deaths, contributed to 60.1%, 64.1% and 69.5% of all deaths in the eastern, central and western regions, respectively, during 2009–2018. The proportion of preterm neonatal deaths due to immaturity was 50.0%, 57.9% and 63.8% in 2017–2018, with meaningful reductions of 33.2%,18.7% and 16.5% compared with 2009–2010 in the eastern, central and western regions, respectively (P < 0.05). Congenital abnormalities and birth asphyxia contributed to 10.7% and 12.2%, 9.0% and 14.9%, 5.5% and 12.7% during 2009–2018, in the eastern, central and western regions, respectively. The proportion of preterm neonatal deaths due to congenital abnormalities increased significantly from 8.8%, 7.6% and 4.1% in 2009–2010 to 18.4%, 9.2% and 6.0% in 2017–2018, in in the eastern, central and western regions, respectively (P < 0.05). The proportion of preterm neonatal deaths due to birth asphyxia increased significantly from 7.1%, 10.9% and 10.0% in 2009–2010 to 10.1%, 18.7% and 13.5% in 2017–2018, in in the eastern, central and western regions, respectively (P < 0.05) (Table 2).

**Table 2:** Proportion of the causes of preterm neonatal deaths among the three regions in China, 2009–2018.

	2009–2010	2011–2012	2013–2014	2015–2016	2017–2018	2009–2018		
						%	χ^2^	P
Eastern regions								
Pneumonia	11(2.2%)	24(5.3%)	11(2.4%)	11(6.6%)	26(4.2%)	86(4.1%)	38.0	0.00
Congenital abnormalities	43(8.8%)	39(8.8%)	40(8.5%)	40(8.9%)	35(18.4%)	218(10.3%)	83.1	0.00
Immaturity	364(74.9%)	274(61.8%)	277(59.0%)	277(55.0%)	213(50.0%)	1297(61.1%)	444.4	0.00
Birth asphyxia	34(7.1%)	54(12.2%)	74(15.8%)	74(15.5%)	60(10.1%)	256(12.1%)	77.0	0.00
Other infectious diseases	9(1.9%)	26(5.8%)	14(2.9%)	14(3.8%)	15(5.1%)	81(3.8%)	33.2	0.00
Other conditions	25(5.1%)	27(6.1%)	53(11.4%)	53(10.2%)	39(12.2%)	185(8.7%)	74.1	0.00
Central regions								
Pneumonia	29(3.9%)	28(3.7%)	32(6.1%)	32(4.6%)	18(4.5%)	124(4.4%)	31.5	0.00
Congenital abnormalities	58(7.6%)	58(7.6%)	57(10.7%)	57(9.7%)	37(9.2%)	247(8.7%)	9.2	0.42
Immaturity	557(73.5%)	532(69.9%)	329(61.9%)	329(55.5%)	210(59.7%)	1870(65.9%)	74.8	0.00
Birth asphyxia	83(10.9%)	89(11.7%)	71(13.3%)	71(20.0%)	76(18.7%)	395(14.0%)	49.8	0.00
Other infectious diseases	2(0.2%)	26(3.4%)	9(1.7%)	9(2.9%)	11(2.6%)	59(2.1%)	45.7	0.00
Other conditions	29(3.9%)	28(3.7%)	33(6.3%)	33(7.3%)	28(5.3%)	139(4.9%)	26.0	0.00
Western regions								
Pneumonia	41(3.2%)	46(3.6%)	54(4.1%)	54(6.0%)	73(4.9%)	265(4.3%)	21.9	0.01
Congenital abnormalities	52(4.1%)	62(4.8%)	82(6.2%)	82(6.3%)	76(6.0%)	334(5.4%)	24.3	0.00
Immaturity	978(76.4%)	963(74.4%)	897(67.7%)	897(65.2%)	791(63.8%)	4292(69.8%)	89.8	0.00
Birth asphyxia	128(10.0%)	14(11.1%)	182(13.7%)	182(15.3%)	186(13.5%)	780(12.7%)	35.5	0.00
Other infectious diseases	8(0.6%)	14(1.1%)	16(1.2%)	16(1.8%)	22(4.3%)	105(1.7%)	94.9	0.00
Other conditions	73(5.7%	65(5.0%)	95(7.1%)	94(5.4%)	65(7.5%)	375(6.1%)	25.2	0.00

**Fig 2:**
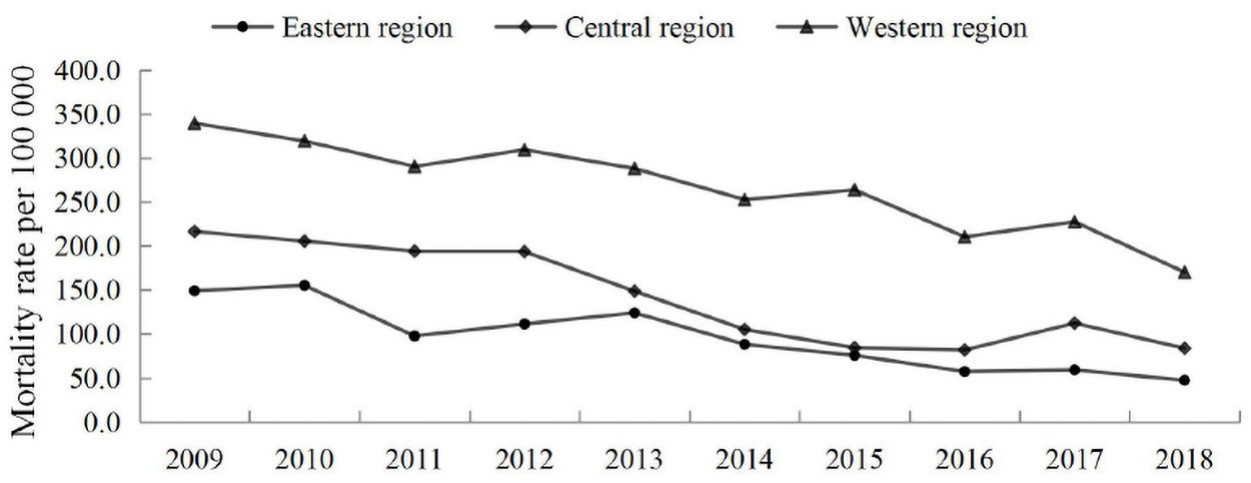
Preterm neonatal mortality rates ascribed to immaturity among the three regions in China, 2009–2018.

The preterm neonatal mortality rate due to immaturity dropped from 149.2, 216.5 and 339.5 in 2009 to 47.4, 83.8 and 170.1 per 100 000 live births in 2018, giving an AADR of 12.1%, 11.6% and 6.3% in the eastern, central and western regions, respectively, during the studying period (Fig 2). The preterm neonatal mortality due to immaturity among the three regions varied: mortality was highest in the western region, moderate in the central regions and lowest in the eastern regions. The adjusted RR for preterm neonatal death with immaturity between the central and eastern regions fluctuated but generally increased during this period. After adjustments were made for residency, the 95% CI of the RR increased from 1.6 to 2.2 and 2.3 to 3.9 for the central and western regions, respectively, during 2009–2018.

In total, 55.1%, 49.8% and 43.6% of preterm neonatal deaths occurred at gestational age <32 weeks in the eastern, central and western regions, respectively, during 2009–2018. The percentage of preterm neonatal deaths at gestational age <32 weeks increased from 54.5%, 46.2% and 39.4% in 2009–2010 to 56.2%, 50.3% and 55.2% in 2017–2018 in the eastern, central and western regions, respectively, with a significant increase only in the western regions. In 2009–2018, the proportion of preterm neonatal deaths at 32-33weeks and 34–36 weeks were 34.1% and 13.5%, 19.3% and 32.4%, 19.9% and 36.7% in the eastern, central and western regions, respectively. (Table 3).

**Table 3:** Proportion of gestational age of preterm neonatal deaths among the three regions in China, 2009–2018.

	2009–2010	2011–2012	2013–2014	2015–2016	2017–2018	2009–2018		
							χ^2^	P
Eastern regions								
28-31W	243(50.0%)	236(53.1%)	273(58.2%)	185(47.8%)	178(53.2%)	1115(52.5%)	9.9	0.04
32-33W	165(34.0%)	144(32.4%)	157(33.4%)	138(35.5%)	119(35.8%)	724(34.1%)	0.9	1.32
34-36W	78(16.0%)	64(14.5%)	39(8.4%)	65(16.7%)	37(11.0%)	284(13.4%)	15.9	0.00
Central regions								
28-31W	336(44.3%)	348(45.7%)	279(52.5%)	200(52.8%)	204(50.6%)	1367(48.2%)	14.9	0.00
32-33W	167(22.0%)	151(19.9%)	86(16.2%)	74(9.6%)	70(17.2%)	549(19.4%)	8.1	0.09
34-36W	255(33.7%)	262(34.4%)	166(31.3%)	105(27.6%)	130(32.2%)	918(32.4%)	6.3	0.18
Western regions								
28-31W	489(38.2%)	534(41.3%)	574(43.3%)	504(41.6%)	571(55.0%)	2672(43.4%)	72.3	0.00
32-33W	255(19.9%)	266(20.5%)	283(21.4%)	244(20.1%)	175(16.8%)	1223(19.9%)	8.3	0.08
34-36W	536(41.9%)	494(38.2%)	468(35.3%)	465(38.3%)	293(28.2%)	2256(36.7%)	49	0.00

**Fig 3:**
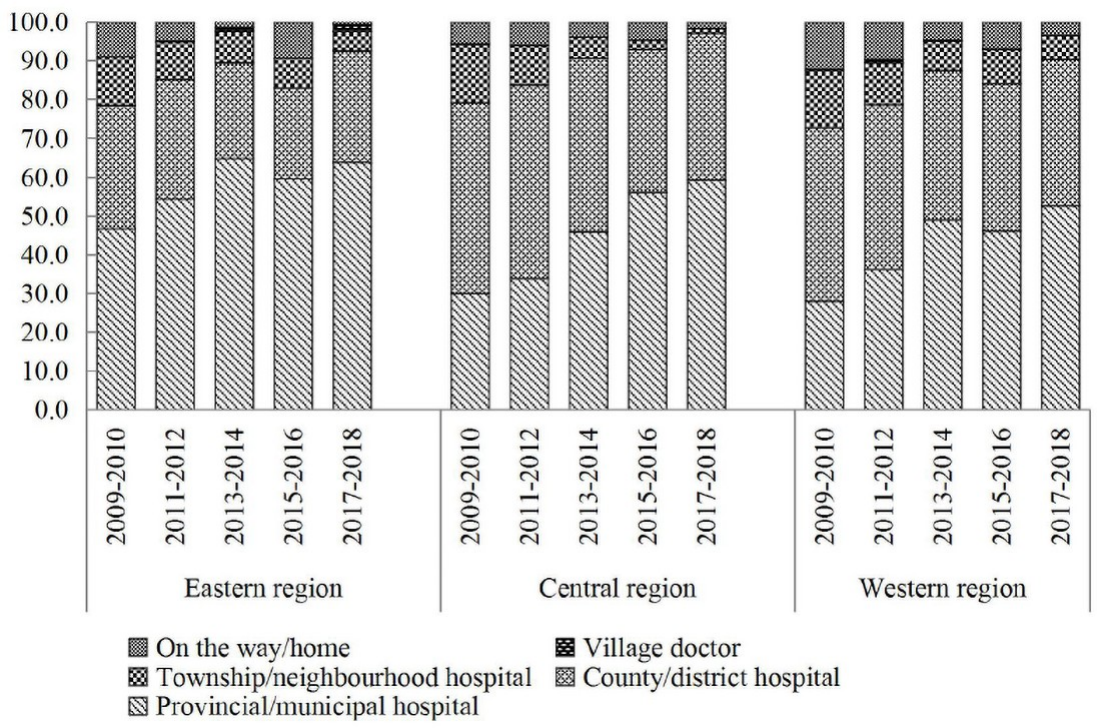
Proportion of location of preterm neonatal infants among the three regions in China, 2009–2018.

There is an obvious increase in the number of preterm neonatal infants born at provincial/municipal hospitals. Preterm neonatal infants born at provincial/municipal hospitals accounted for 63.7%, 59.3% and 52.7% of preterm births in 2017–2018, which increased from 46.7%, 30.1% and 27.9% in 2009–2010 in the eastern, central and western regions, respectively. Accordingly, the percentage of preterm infants born at county/district hospitals significantly decreased during this time, from 31.9%, 49.1% and 44.9% in the eastern, central and western regions to 28.9%, 37.8% and 37.8%, respectively. The proportion of neonatal deaths of preterm babies delivered at home or on the road was higher in western regions than in the central and eastern regions (Fig 3).

## Discussion

Despite substantial progress, there is a significant increase in the proportion of preterm infants among neonatal deaths, which explains 50% of neonatal deaths in 2009–2018. Immaturity, congenital abnormalities and birth asphyxia were the leading causes of preterm neonatal deaths. Overall, the preterm neonatal mortality of immaturity decreased significantly in China during 2009–2018. It is worth noting the regional disparities in the characteristics of preterm neonatal deaths. The rate of preterm neonatal mortality due to immaturity and the proportion of preterm neonatal deaths with gestational age <32 weeks were highest in the western region, followed by the central region.

Preterm birth has been one of the major causes of neonatal death in China. Based on our estimate, close to 44–50% of all neonates died directly or indirectly from preterm birth in 2009–2018, with a significant increase in all regions, compared with 33.6–40.9% in 2003–2008 [19]. However, preterm infants were only linked to 35.2% of infant deaths in the United States [20]. In the recent two decades, the prevalence of preterm birth has significantly increased [21], especially after the Two-child Policy was implemented in China [22]. The rate of preterm birth increased by 23.9% in the past three decades, from 5.4 in 1990–1994 to 7.0 per 100 live births in 2016 [23]., Increasing preterm birth rates are affected by multiple factors, including an increasing proportion of pregnant women over 34 years old, greater use of assisted reproduction technology and increasing number of multiple births [24]. In China, the birth rate in women aged 35 to 39 increased from 8.7‰ in 2004 to 17.0‰ in 2014, and in the 40–44 age group, the rate raised from 1.8‰ to 4.0‰ [25]. The ratio for advanced pregnancies was about 31% of the total pregnancies after the universal two child policy announcement [26]. In addition, assisted reproduction technology (ART) in China has been developing rapidly and dramatically since the first ‘test-tube’ baby was successfully born [27]. One nationwide survey demonstrated that total ART cycle procedures of in vitro fertilization increased from 78 002 during 1981–2004 to 393 538 during 2005–2011 [27]. Furthermore, with the widespread development of ART, the incidence of monozygotic twins has increased significantly [28]. Therefore, the burden of preterm birth was probably the underlying cause for the increase of neonatal mortality rate.

In our study, immaturity, congenital abnormalities and birth asphyxia were the leading causes of preterm neonatal deaths, which contributing to 80% of preterm neonatal deaths. Immaturity, the list leading cause of preterm infant deaths, still explains 50–60% of deaths in 2017–2018, although there is a significant reduction in all geographical regions during the study period. We found remarkable strides in reducing preterm neonatal mortality due to immaturity in all regions in 2009–2018. These decreases can be attributed to Chinese fast socioeconomic growth. In 2018, the per capital disposable income of Chinese residents was ¥28228, with an increase of 748% over the past decade [29]. The proportation of the annual spending on healthcare per capita has also increased from 6.4% in 2000 to 7.8% in 2018 [26]. In addition, China attaches great importance on maternal and child survival and health could be another important contributor [30]. For example, the Chinese government has promulgated the National Program of Action for Child Development in China every decade since 1990s [31], the National Health Commission of People’s Republic has issused the Opinions on Strengthening the Reform and Development of Children’s Medical and Health Services in 2016 [32].

However, the regional disparities among these regions still existed. In our study, the rate of preterm neonatal mortality due to immaturity in the western region was more than two times that of the developed eastern region, and this gap has increased since 2009. Overall, because of inadequate health service infrastructures and low health expenditure due to economic underdevelopment are thought to have resulted in limited access and use of health services in mothers and children, which led to inequity preterm survival status in the western region [33, 34] The number of hospitals and primary care institutions per 1000 km^2^ in the eastern zone was 8 and 2 times more than that in the western zone, respectively [35]. In China, most quality health resources are concentrated in hospitals, especially in tertiary hospitals, of which 46.7% are located in the eastern areas [36]. In addition, preterm birth rate varied by region with the West having the highest occurrence, of which the preterm birth rate was 7.3% in the Northwest and 7.0% in the Southwest [23]. Obviously, these regional disparities should be systematically addressed in the SDG era. Therefore, maternal and child survival policy, programs, and resources targeting the western region should receive more attention from all levels of government in China.

In our study, most preterm neonatal deaths occurred with gestational ages at birth of <32 weeks in all regions. This is similar to findings in high-income countries such as the US and Canada [37]. We also found that there was an increase in preterm neonatal deaths of infants with gestational ages at birth of <32 weeks in the three geographical regions. The risk of prematurity related morbidities, such as bronchopulmonal dysplasia(BPD), necrotising enterocolitis(NEC), intraventricular haemorrhage (IVH), periventricular leukomalacia (PVL), retinopathy of prematurity (ROP), increases with decreasing gestational age [38]. The risk of mortality increases proportionally with decreasing gestational age. The mortality rate of gestation age at 30 weeks, 31weeks, 32 weeks, 33 weeks, 34 weeks, 35 weeks is 39.3, 31.3, 23.2, 19.1, 13.47 and 9.1 per 1000 live births [39]. In China, the very preterm infants (gestational age <32 weeks) increased significantly compared with 2002 [40]. In addition, with the medical development, the treatment ability of preterm infants has improved, especially for preterm infants with a gestational age of 32 to 36 weeks, and relevant policies and actions, such as improving local obstetric infrastructure in hospitals in the county and township, have been effective. However, this reflect that the treatment capacity of country/district hospitals for these preterm neonatal infants with gestational ages at birth of <32 weeks is still insufficient.

According to our study, there is an obvious increase in the number of preterm neonatal infants born at provincial/municipal hospitals from 2009 to 2018. In 2017–2018, over half of preterm neonatal infants were born at provincial/municipal hospitals in the three geographical regions. This suggests that the accessibility and quality of preterm neonatal infant care has improved significantly, especially in the western region. However, approximately 45% of preterm neonatal infants are born at country/district hospitals, township/neighborhood hospitals, and village clinics. Moreover, for preterm neonatal infants <32 weeks, the treatment capacity of country/district hospitals for these preterm neonatal infants is still insufficient. Therefore, the management of high-risk pregnant women should be strengthened; preterm neonatal infants <32 weeks should be transported in utero and delivered at higher-level provincial/municipal hospitals. Preterm neonatal infants born at provincial/municipal hospitals led to lower preterm neonatal mortality in the eastern region than in the coastal and western regions. This indicates that certain procedures can facilitate the improvement of regional disparities in preterm neonatal mortality.

Neonates mortality and morbidity from preterm birth can be reduced through effective interventions delivered to the mother before or during pregnancy, and to the preterm infant after birth [41]. Interventions can be directed at all women for primary prevention, such as: reducing the risk of preterm birth (smoking cessation programme), minimizing the risk in women with known risk factors (progestational agents, cervical cerclage) [42]. However, the most beneficial set of maternal interventions are those that are aimed at improving outcomes for preterm infants when preterm birth is inevitable (antenatal corticosteroids, magnesium sulfate and antibiotic prophylaxis) [41]. In addition, a substantial amount of new evidence has emerged on preterm newborn interventions in recent years, including the use of Kangaroo mother care (KMC) [43], plastic wraps [44], continuous positive airway pressure [45] and surfactant therapy. Further improvements in neonatal survival will require a higher proportion of deliveries occurring in well-equipped facilities with high-quality care [46]. Last, reductions in mortality rates in infants that were born preterm have been driven largely by appropriate policy changes. Therefore, primary and secondary prevention of preterm birth should receive more attention from the Chinese government, priority interventions should be region-specific, depending on the availability of economic and health care resources. Firstly, the local communities should strengthen pregnancy health care and health knowledge. Secondly, the local governments need to strengthen the construction of RNTN (Regional Neonatal Transport Network), Neonatal Intensive Care Unit (NICU) and ensure the applying of professional medical staff and newborn rescue equipment for neonatal transport. Thirdly, local health departments can regularly organize multi-level, especially provincial, prefecture-level and district-level) newborn health training programs. Considerable investments in terms of training and health infrastructure are needed to enable skilled birth attendants to deliver lifesaving interventions, especially during delivery and the first week of life.

There are some limitations in our study. First, our data were not related to parental situation or family background, which prevented us from exploring the impact of these factors on preterm neonatal mortality. Second, we cannot assess preterm neonatal mortality among infants with different gestation because there are no live births at different gestation. Third, our analysis does not reveal the distribution of risk factors for preterm birth, largely because there are no cause of preterm birth in U5CMSS.

## Conclusions

To conclude, to advance neonatal survival and achieve the SDG goals, it is important to focus on preterm survival. During the study period, there is a significant increase in the proportion of preterm infants among neonatal deaths, which explains 50% of neonatal deaths. With the joint efforts of the economic development and the governmental policies and strategies, the preterm neonatal mortality rate of China has significantly decreased from 2009 to 2018. It is worth noting the regional disparities in the characteristics of preterm neonatal deaths. The rate of preterm neonatal mortality due to immaturity and the proportion of preterm neonatal deaths with gestational age <32 weeks were highest in the western region, followed by the central region. Therefore, preterm infants remain a priority for intervention to reduce neonatal mortality and to achieve SDG 3.2, especially in the western region. Our study may provide basic information and may help direct the Chinese government to further decrease the total neonatal mortality rate and reduce geographical disparities by increasing healthcare accessibility through the formulation of related policies.
